# On Certainties and Probabilities

**Published:** 1917-11-03

**Authors:** 


### The Hospital

The Workers' Newspaper of Administrative Medicine and Institutional Life, Administration, National Insurance and Health.

No. 1639, Vol. LXIII. SATURDAY, NOVEMBER 3, 1917. PRICE ONE PENN

### ON CERTAINTIES AND PROBABILITIES.

A reproach sometimes urged against a scientific training is that it breeds an incredulous attitude towards any proposition which cannot be accommodated to a formula or demonstrated in a laboratory. Here in the student's experience is the test which asserts itself as the daily criterion, and almost inevitably he is inclined, in greater or less measure, to carry it into the outside world and to apply it to the questions which there confront him. Let it be admitted that the record is a correct one, and it may still be claimed that the extension of the scientific standard of accuracy to the propositions of daily life and conversation is very far from a pure misfortune. Loose thinking and facile phrases and conventional conclusions are all too abundant in both our reading and our writing, not to speak of the practice of what is politely called our conversation. To introduce into this easy and indefinite and commonplace atmosphere some precise standard of truth and accuracy may indeed produce some startling effects, but it can hardly fail to have a measure of intellectual and disciplinary value for the one who practises it. His range of certainty may be narrowed, but it will stand on a more confident basis. As a competitor in the art of small-talk as this is cultivated in social circles the student disciplined by science will be easily outdistanced by less restrained tongues. But he may be content to lose this race, knowing that he possesses more secure triumphs. Though he cannot, like the majority of his neighbours, produce a ready opinion on each and all of the various subjects which occupy the moment, he has certain safe retreats where he cannot be shaken. And he is never in danger of mistaking tastes or prejudices or conventions or party cries or shibboleths for reasoned opinions or intellectual convictions. There are many subjects on which he must perforce confess himself ignorant and destitute of "views." But in a certain limited field he feels himself on sure ground and is consoled by the conviction of intellectual honesty. Hence even if, as it is said, his training develops incredulity, this is not without advantages to himself, however much it may isolate him from his more conversational acquaintances.

Yet here, as in most directions, there may be an error of excess. The rigid standard of scientific truth and mental honesty does, without question, make for clear thinking and accuracy of statement, and in the world of opinion and of belief it would be an ill-service to try to abate its claims. All the same it is equally true that in many of the affairs of life something less than this has to be accepted, at least as a basis for action and decision. Probability rather than certainty has to be accepted as the only available guide. To quarrel with such a condition is to quarrel with the constitution of the world in which we live, and of which, somehow or other, we have to make the best we can. It must, therefore, sometimes happen that in the school of life the man who waits for certainty will wait for something which will not arrive, and in the mean time the opportunity has gone from him. The balance of evidence, and not actual demonstration, must be accepted as the determining factor, and prudent action must be willing to rest itself here and not to fail because the more confident sanction is lacking. Obviously this is not to say that evidence is to be disparaged. On the contrary, it is to urge that all available evidence ought to be collected and reviewed. But when this has been done the time for decision has arrived, and action must be taken with a courageous and firm will. To hesitate at this course or to decline it is to fail to realise those conditions of life which just as clearly announce that actions must often be directed by probabilities as they proclaim that "opinions" apart from evidence are little more than idle and empty chatter. In so far as the popular reproach of science is justified, it is in a failure to recognise this distinction that its sanction is to be found. Right and proper it is that the severe standard of scientific doctrine should be extended into realms of opinion which stand outside the laboratory. But it is an equal claim that the teachings of the school of practical life shall be accepted and that the balance of probability shall be regarded as a sufficient justification and demand both for wise decision and for prompt action. To confound these two spheres does afford a certain appearance of justice to those who urge that the mental atmosphere provided by scientific work and study is an ill-preparation for the realities and emergencies of practical life. There is without question a region in which wise men hesitate and proclaim their ignorance, while more adventurous spirits enter with cocksure confidence. But not less certainly there is another region on the threshold of which is written as a warning—he who hesitates is lost.

A scheme of training which would include both of the influences here mentioned might well be proposed as an almost ideal instrument of education. In our judgment it is hardly too much to say that medicine may make this profession. Here opinion is taught to be free, and at the same time is urged to seek no other sanction than that of evidence. To this test the words even of the most authoritative must be content to come, and by such an appeal every thesis must be appraised. Yet practical issues to be settled by a judgment on the probabilities as these are decreed by evidence are continually presenting themselves, and they will not await academic certainty. Thus while the mind is preserved from the pressure and restriction of authority there is much occasion for prompt decision and ready action. The chastening influence of science on mental sluggishness and sloppiness is ever present, but in equal measure is the pressure to meet with efficiency the emergency of time and the event.

